# Supplementary material for: Phosphatase specificity influences phosphorylation timing of CDK substrates during the cell cycle
Source: Nat Commun. 2025 Nov 24;16:11604. doi: 10.1038/s41467-025-66547-5 (PMC12749991; doi:10.1038/s41467-025-66547-5)
Supplement: Supplementary file 1 — Supplementary Information [file 41467_2025_66547_MOESM1_ESM.pdf]

Supplementary Information

Phosphatase specificity influences phosphorylation timing of CDK substrates during the cell cycle

Theresa U. Zeisner (ORCiD: 0000-0002-2898-289X)[1,4,*], Tania Auchynnikava[1,2], Emma L. Roberts[1], Paul Nurse[1,3]

[1]Cell Cycle Laboratory, The Francis Crick Institute, London, UK
[2]Proteomics Platform, The Francis Crick Institute, London, UK
[3]Laboratory of Yeast Genetics and Cell Biology, Rockefeller University, New York, NY, USA
[4]Current address: Research Institute of Molecular Pathology (IMP), Vienna BioCenter (VBC), 1030 Vienna, Austria
[*]Correspondence: theresa.zeisner@imp.ac.at

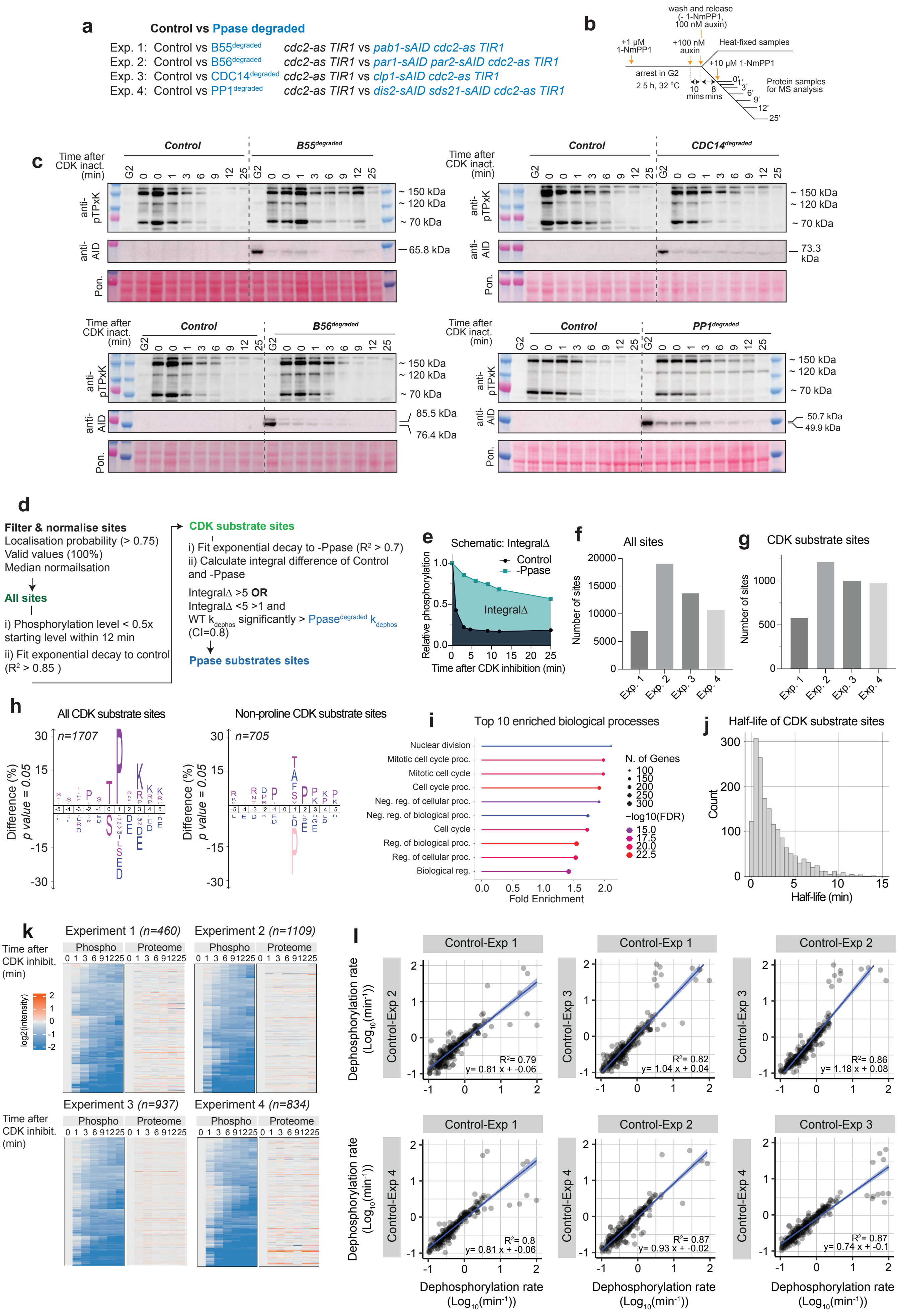

Supplementary Figure 1: Identification of *in vivo* CDK substrate sites

a List of strains used in each of the four phosphoproteomic experiments. **b** Detailed schematic of phosphoproteomic experiment. **c** Western blots of phospho-CDK substrates and sAID-tagged phosphatases after release from G2 arrest. Numbers indicate the time in minutes after CDK inactivation at peak mitosis. Two samples were taken simultaneously at timepoint 0. Membranes were probed with an anti-pTPxK antibody and anti-AID antibody. Ponceau-S stain was used for total protein normalisation. Uncropped blots are supplied in the Source Data file. Blots representative of *n=3* biological repeats for B55^{degraded}, *n=2* for B56^{degraded}, CDC14^{degraded}, and *n=1* for PP1^{degraded}. **d** Criteria for classifying phosphatase substrates (see also Methods). **e** Schematic plot showing the difference of integrals (from 0-25 mins after CDK inhibition), used to classify phosphatase substrates. **f-g** Bar graph representing the number of **f** all phosphosites detected in the four phosphoproteomic experiments after filtering and **g** number of identified CDK substrate sites. **h** IceLogo representation of over- and underrepresented amino acid residues surrounding the phosphorylation sites of all identified CDK substrate sites (left, *n=1707* sites) and all CDK substrate sites which do not contain a Proline at the +1 position (right, non-consensus sites, *n=705*)[1]. **i** GO-enrichment of CDK substrate sites, showing the 10 most enriched biological processes using ShinyGO[2]. **j** Histogram of half-life (min) of all identified CDK substrate sites. **k** Heatmap visualising the changes in phosphosites (left) and the respective proteins (right) upon inactivation of CDK activity. Only sites which are classified as CDK substrate sites and for which the corresponding protein was detected in the proteome are plotted. **l** Pairwise linear regressions of dephosphorylation rates determined for phosphosites in the control conditions of the 4 different phosphoproteomic experiments.

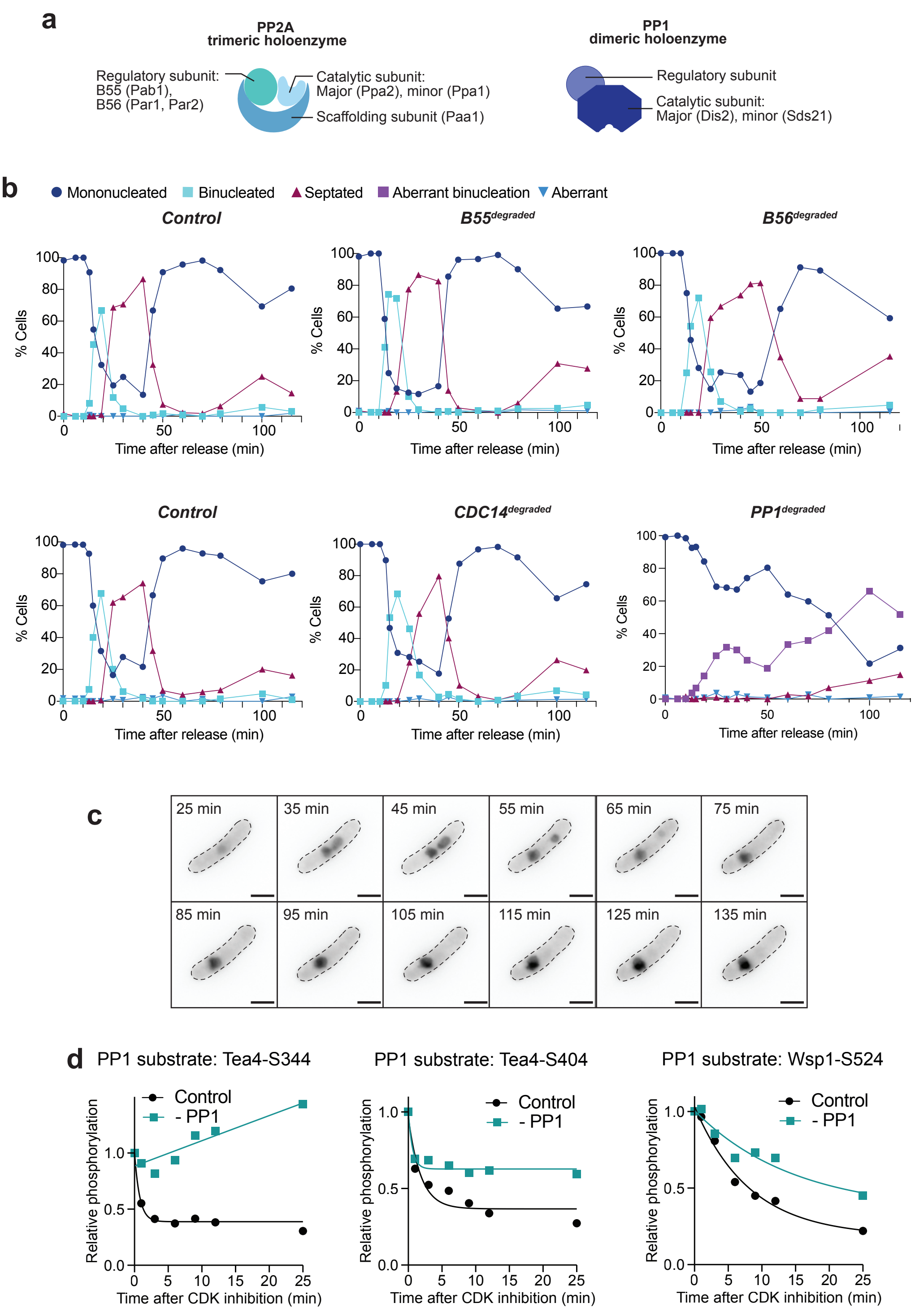

Supplementary Figure 2: Rapid degradation of phosphatases using the AID system

a Schematic of PP2A and PP1 holoenzyme subunits. **b** To follow mitotic progression after release from a G2 arrest in the presence and absence of phosphatases, binucleation and septation indices were quantified from DAPI- and calcofluor-stained fixed cells. Timepoint zero represents the point at which G2-arrested cells were washed and released into 1-NmPP1-free media. At least 100 cells were counted per timepoint (exact n-numbers given in Source Data). Representative of *Control: n=8, B55*^{degraded}*: n=4, B56*^{degraded}*: n=2, CDC14*^{degraded}*: n=3, PP1*^{degraded}*: n=1* independent biological experiments. Degradation of PP2A-B56 regulatory subunits Par1 and Par2 led to broader septation peaks and a delay in cytokinesis, consistent with Par1 promoting cytokinesis after a prolonged metaphase arrest[3] and *par1Δpar2Δ* double mutants showing a higher incidence of double septa and misplaced septa[4]. Degradation of CDC14 resulted in a short delay in the start of septation, consistent with CDC14 playing a role in cytokinesis via the septation initiation network[5,6]. Degradation of both catalytic subunits of PP1 (Dis2 and Sds21) led to a severe binucleation defect (aberrant binucleation; see also Supplementary Fig. 2c), consistent with PP1 activity being required for accurate chromosome segregation[7]. **c** Example timelapse images of SynCut3-mCherry fluorescence (Cut3 is a Condensin subunit and is used here as a nuclear marker) in cells after PP1 degradation using the AID system. MaxZ projections of fluorescent images are shown, with dark areas indicating higher fluorescence. Cells were arrested in G2 using 1 μM 1-NmPP1, PP1 was degraded using auxin and cells were released from the G2 arrest 10 minutes later. Time in images

refers to minutes after release from G2, images representative of n=*132* cells (*n=2* biological repeats). Cells are not viable long-term (data not shown), consistent with a *dis2*Δ*sds21*Δ mutant being lethal[8]. The dashed lines indicate single-cell masks, generated from the bright-field image. Scale bar represents 5 µm. **d** Example PP1 substrates involved in polarised cell growth: the polarity factor Tea4 and the actin-binding protein Wsp1. Plots show relative phosphorylation level in the presence (black) and absence (turquoise) of the phosphatase of interest upon CDK inhibition. Curves are a one-phase exponential decay fitted to the relative phosphorylation level determined by phosphoproteomics.

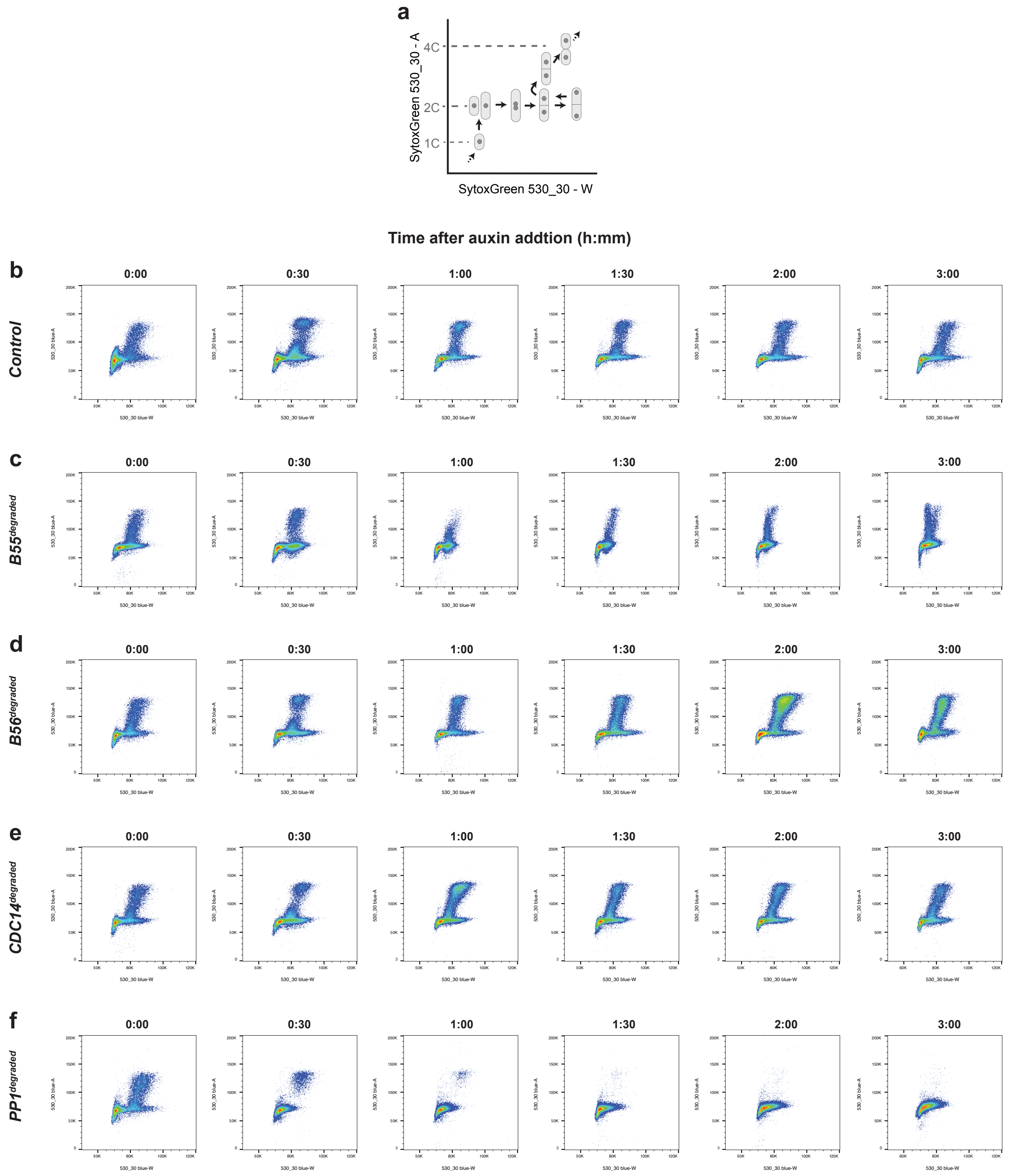

Supplementary Figure 3: Flow cytometry analysis after phosphatase degradation

a Schematic 2D plot of SytoxGreen signal area vs width with respective cell cycle stages indicated. **b-f** 2D plot of SytoxGreen signal area (530_30-A) vs width (530_30-W) to identify cell cycle stages upon degradation of indicated phosphatases using auxin. Time after auxin addition is indicated as h:mm. Upon PP2A-B55 degradation, cells shift to a smaller width, consistent with cells becoming substantially shorter in the absence of PP2A-B55, and nuclei thus being closer together. Upon PP1 degradation, binucleated cells disappear, consistent with cells entering a metaphase arrest in the absence of PP1 activity.

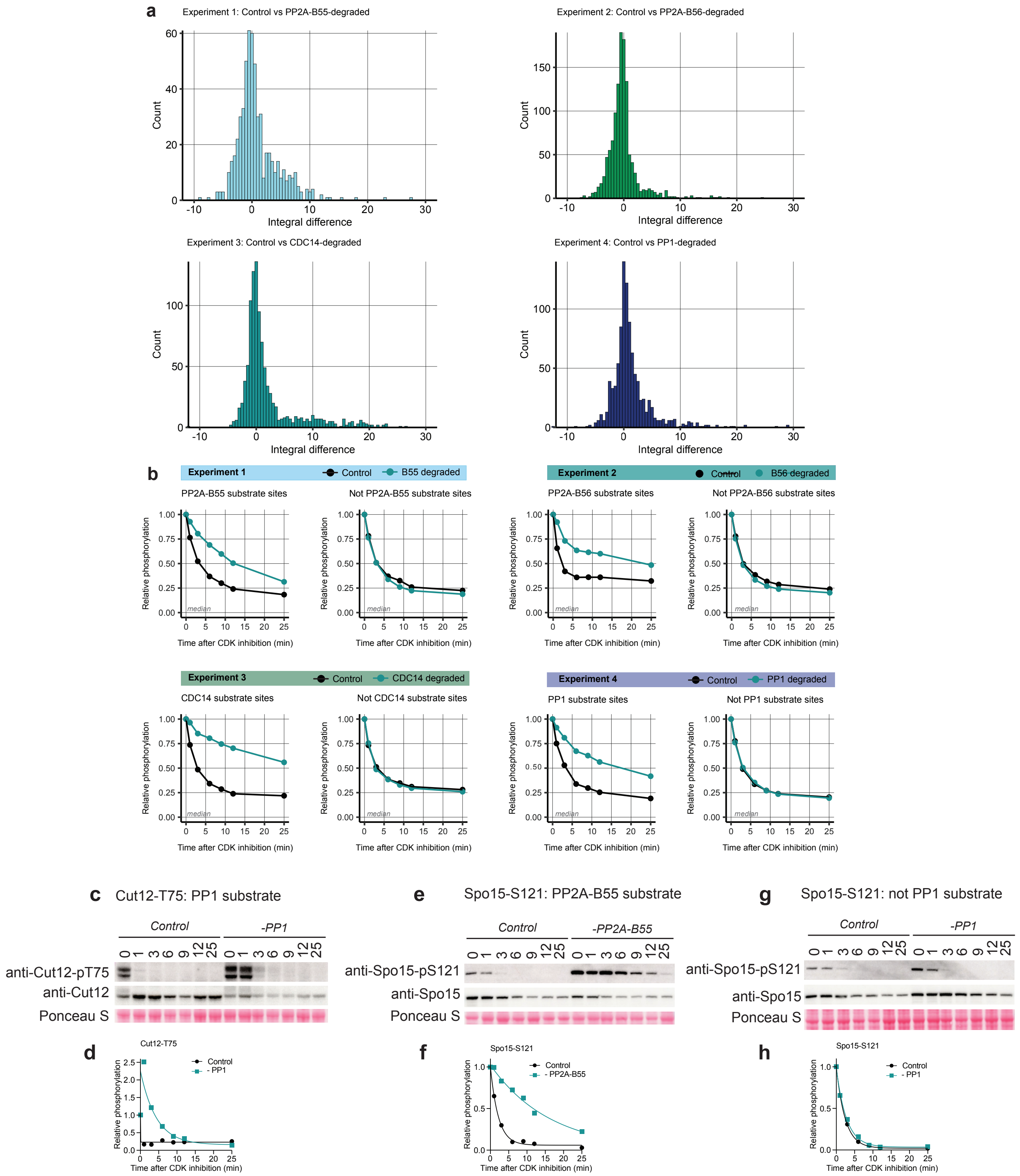

Supplementary Figure 4: Identification of *in vivo* phosphatase substrates

a Histogram of integral difference (explained in Supplementary Fig. 1e) between -Ppase and Control condition in all CDK substrate sites in the four different phosphoproteomic experiments. **b** Median relative phosphorylation of all phosphatase substrates and not phosphatase substrates in the presence and absence of the respective phosphatase. **c-h** Western blots of phospho-Cut12-T75 and phospho-Spo15-S121 after release from G2 arrest in the presence and absence of the indicated phosphatases (**c,e,g**). Uncropped blots are supplied in the Source Data file. Blots representative of *n=2* biological repeats. Numbers indicate the time in minutes after CDK inactivation at peak mitosis. Corresponding phosphoproteomic data for the indicated sites are shown underneath the Western blots (**d,f,h**). Plots show relative phosphorylation level in the presence (black) and absence (turquoise) of phosphatase of interest upon CDK inhibition. Curves are a one-phase exponential decay fitted to the relative phosphorylation level determined by phosphoproteomics.

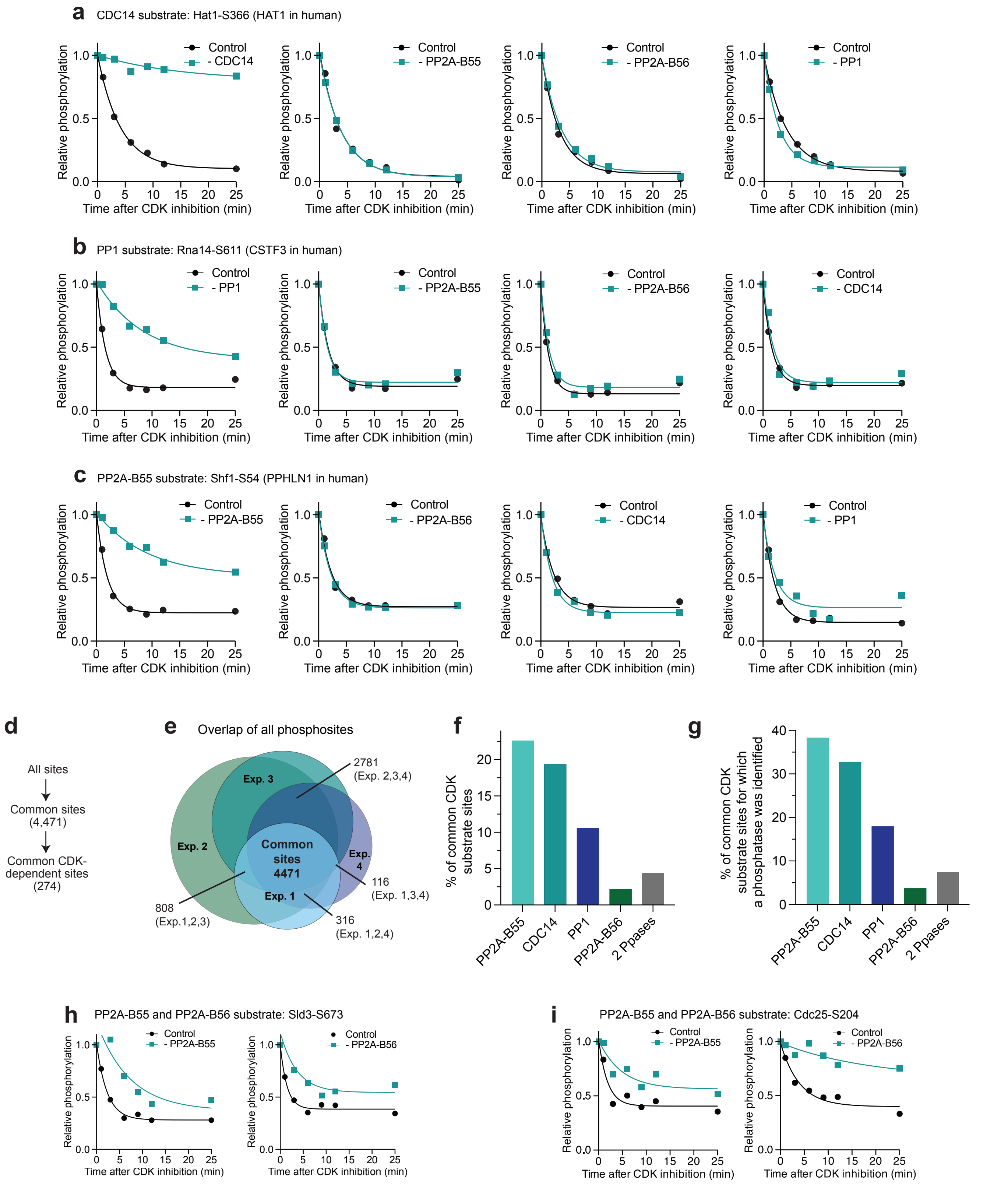

Supplementary Figure 5: Substrate specificity of investigated phosphatases

a-c Examples of phosphatase substrates, which are targeted by just one of the investigated phosphatases. Relative phosphorylation level in the presence (black) and absence (turquoise) of phosphatase of interest upon CDK inhibition. Curves are a one-phase exponential decay fitted to the relative phosphorylation level determined by phosphoproteomics. **d** Schematic of filtering for sites, which are identified in all 4 phosphoproteomic experiments. **e** Venn diagram depicting the overlap of all detected phosphorylation sites in the four experiments. f-g) Bar graph showing the phosphatase substrates as a percentage of **f** all common CDK substrate sites **g** CDK-substrate sites that are targeted by at least one of the investigated phosphatases. **h-i** Example of phosphatase substrates, which are targeted jointly by two of the investigated phosphatases. Relative phosphorylation level in the presence (black) and absence (turquoise) of phosphatase of interest upon CDK inhibition. Curves are a one-phase exponential decay fitted to the relative phosphorylation level determined by phosphoproteomics.

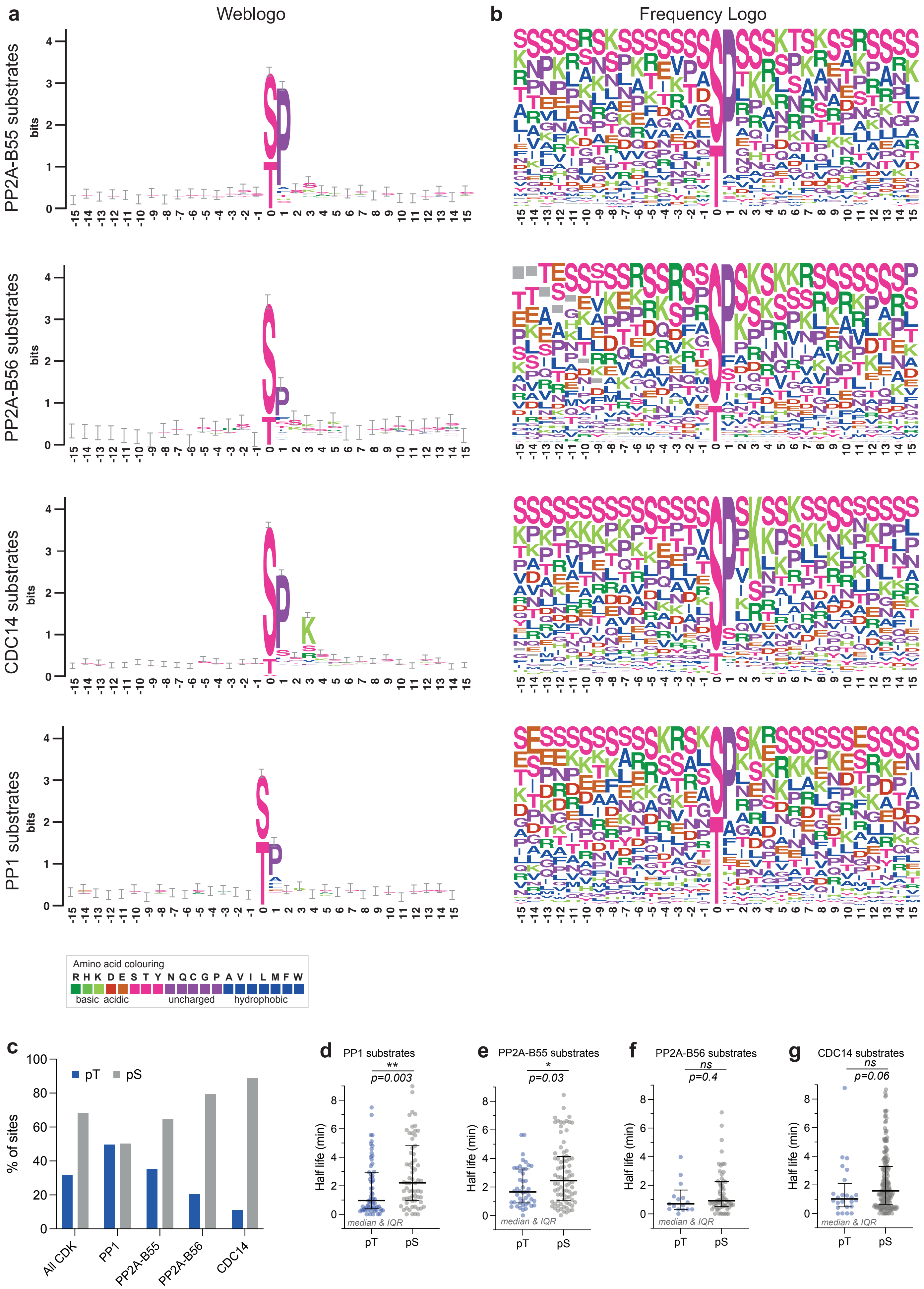

Supplementary Figure 6: Sequence logos of CDK-dependent phosphatase substrates

a-b Amino acid sequences (31 aa) centred on the phosphorylated residue of the identified phosphatase substrates were used to generate Weblogos and frequency logos of CDK-dependent phosphatase substrates[9]. **a** For Weblogos the height of each stack indicates the sequence conservation, whereas height of each amino acid within a stack reflects the relative frequency of that amino acid **b** Frequency logos show the frequency of each residue at a given position. **c** Grouped bar graph representing the percentage of pThreonine and pSerine in CDK substrate sites and CDK-dependent phosphatase substrates. **d-g** Half-life of pThreonine and pSerine CDK-dependent phosphatase substrates, for each of the four investigated phosphatase substrates. Error bars denote the median and IQR. Statistical difference between groups was determined using a Mann-Whitney test (two-tailed). N-numbers are provided in the Source data.

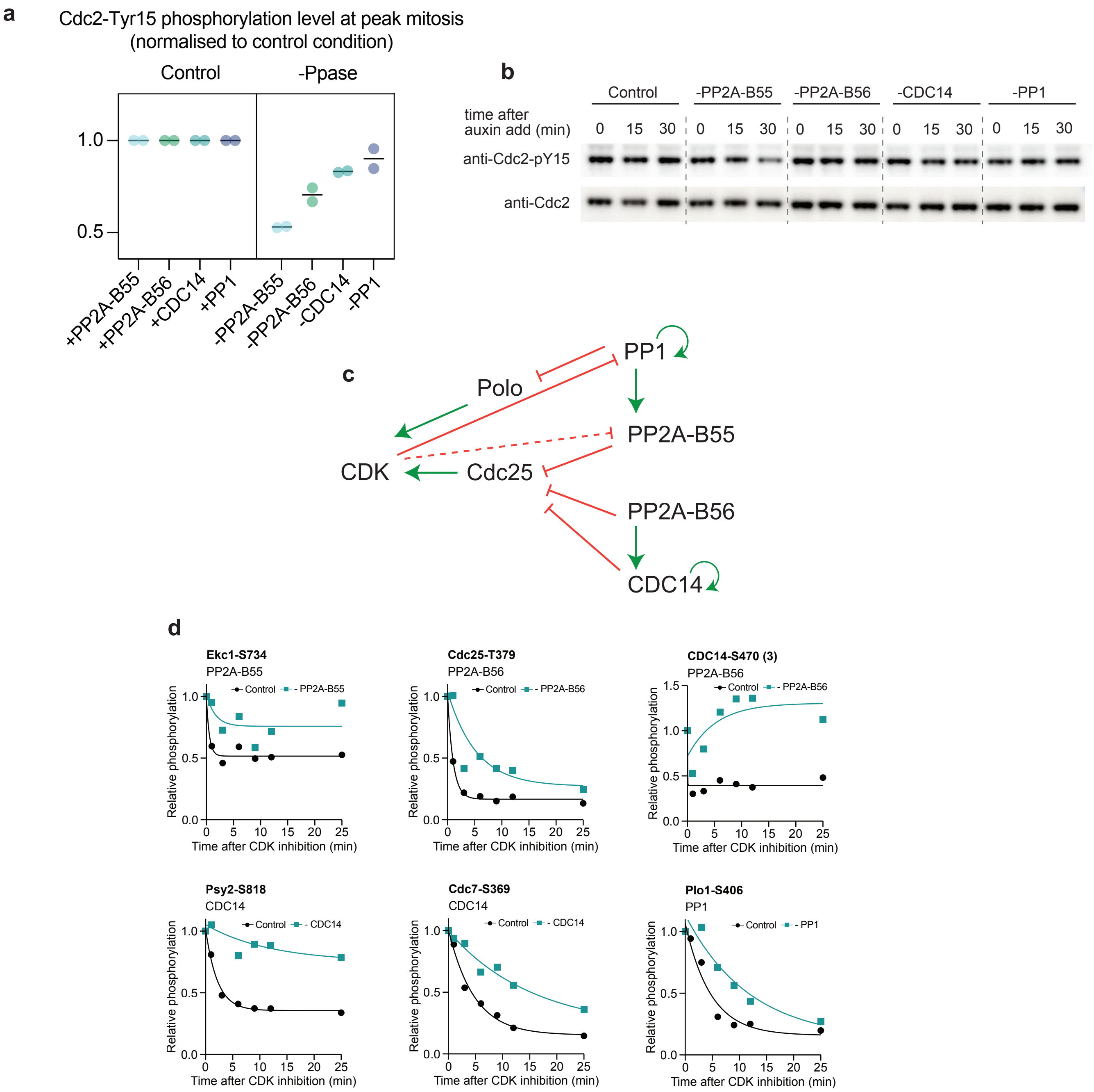

Supplementary Figure 7: Interplay between CDK and phosphatase activity

a Relative phosphorylation level in mitosis of Cdc2-Tyr15 in the presence (control) and absence of the indicated phosphatases. **b** Western blot against phospho-Cdc2-Tyr15 and Cdc2. Cells were treated with 1 µM 1-NmPP1, leading to an arrest in G2, at which point cells were treated with auxin to degrade phosphatases. Numbers above the lanes indicate time after auxin addition. Representative of n=1. **c** Schematic depicting the interplay between CDK and CDK-opposing phosphatases. **d** Example phosphatase substrates identified in the phosphoproteomic dataset, highlighting the interconnectedness between CDK and phosphatase activity regulation. PP2A-B55 dephosphorylates a CDK-site on the PP6 regulator Ekc1, while CDC14 dephosphorylates a site on the PP4 regulator Psy2. Plots show relative phosphorylation level in the presence (black) and absence (turquoise) of phosphatase of interest upon CDK inhibition. Curves are a one-phase exponential decay fitted to the relative phosphorylation level determined by phosphoproteomics

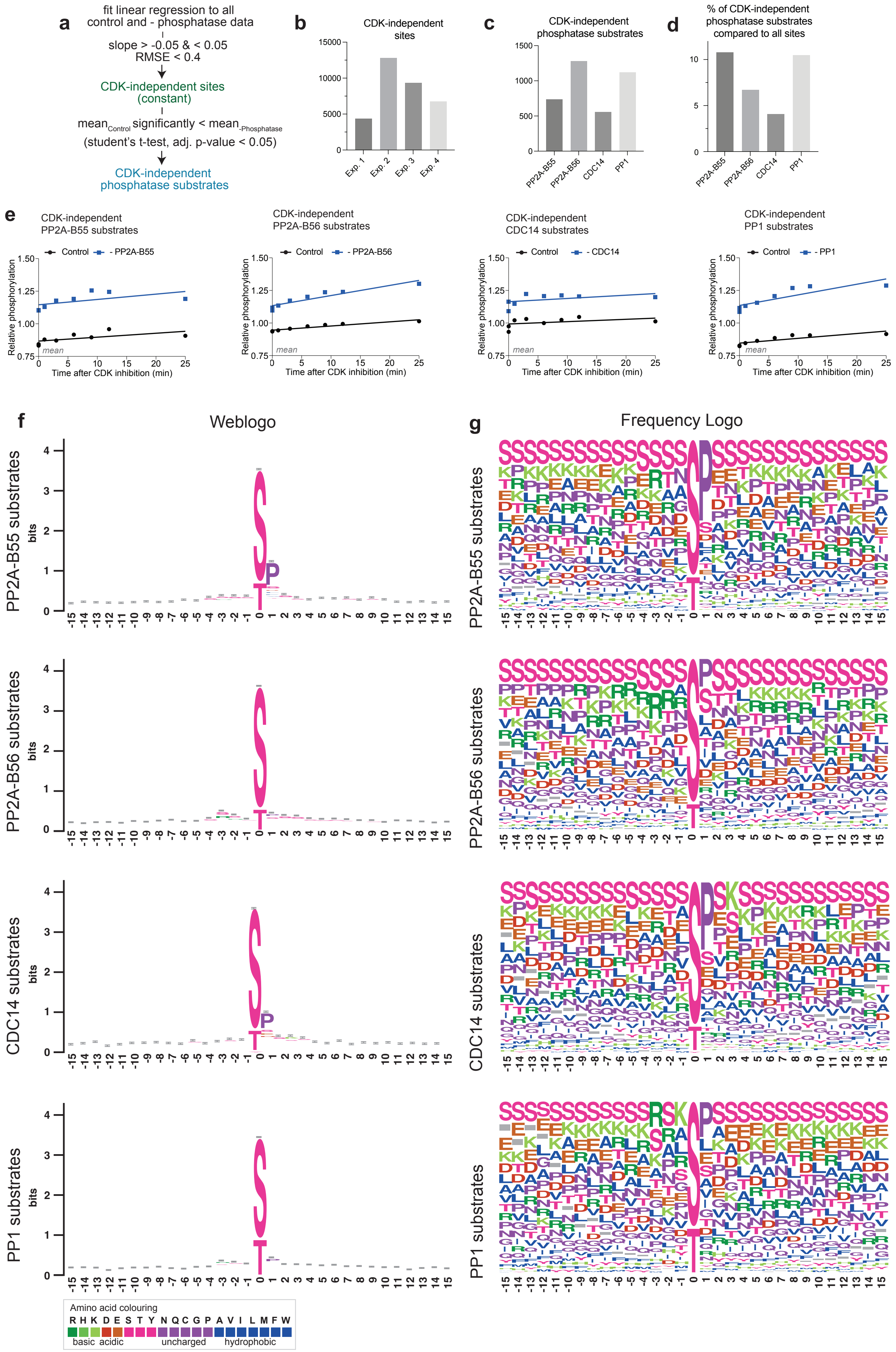

Supplementary Figure 8: Identification of CDK-independent phosphatase substrates

a Criteria to identify CDK-independent sites (constant sites) and CDK-independent phosphatase substrates b-d) Bar graph representing the **b** number of identified CDK-independent sites, **c** number of CDK-independent phosphatase substrates and **d** the percentage of CDK-independent phosphatase substrates of all CDK-independent in the four different phosphoproteomic experiments. **e** Mean phosphorylation of CDK-independent phosphatase substrates for PP2A-B55, PP2A-B56, PP1, and CDC14 in the presence (black) and absence (blue) of the respective phosphatases. A linear model was fitted through the mean points. **f-g** Amino acid sequences (31 aa) centred on the phosphorylated residue of the identified phosphatase substrates were used to generate Weblogos and frequency logos of CDK-dependent phosphatase substrates[9]. **f** For Weblogos the height of each stack indicates the sequence conservation, whereas height of each amino acid within a stack reflects the relative frequency of that amino acid. **g** Frequency logos show the frequency of each residue at a given position.

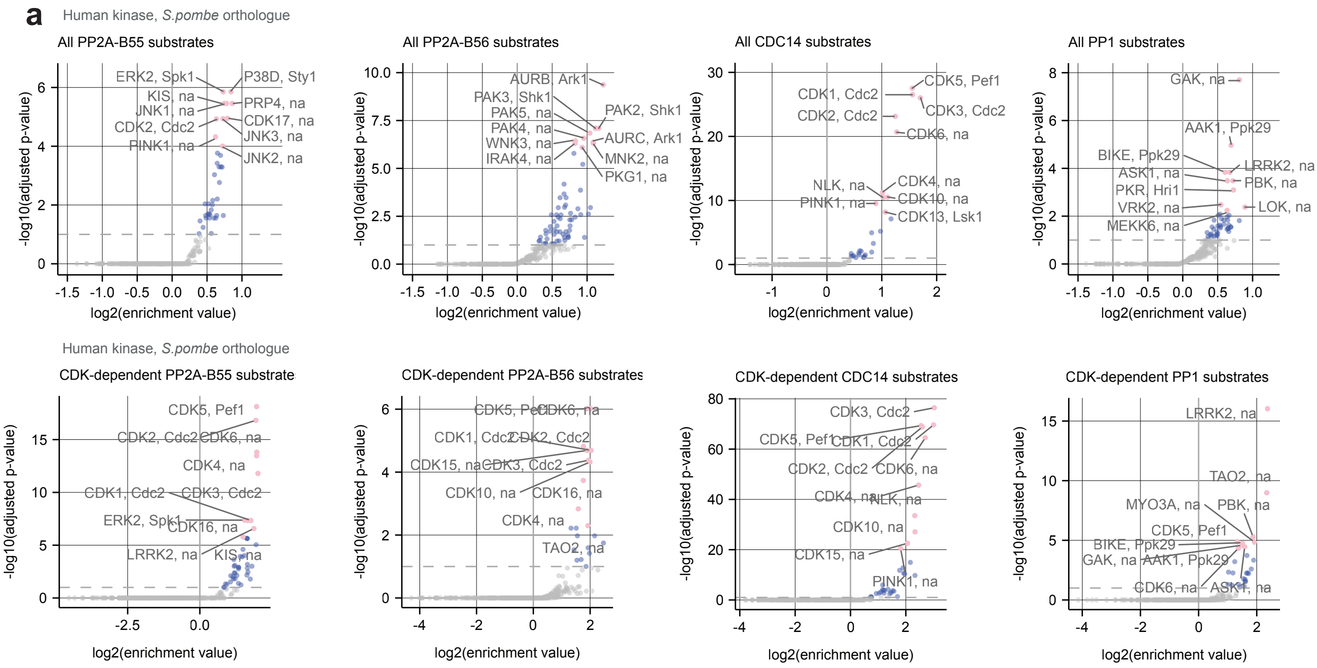

b

Kinase	S. pombe name	Consensus sequence
CDK	Cdc2	[ST]P
Polo	Plo1	[DENY].[ST][FLIYWVM] or SF
Aurora B	Ark1	R.[ST][^P]
NIMA	Fin1	[LMFW][^P][^P][ST][^P]
NDR2	Sid2	R..[S]
DDK	Hsk1	[ST][DE] or [ST][ST][PQ]

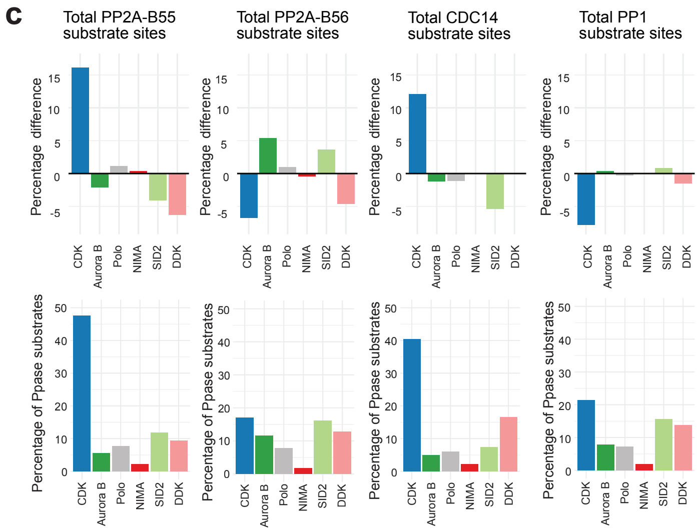

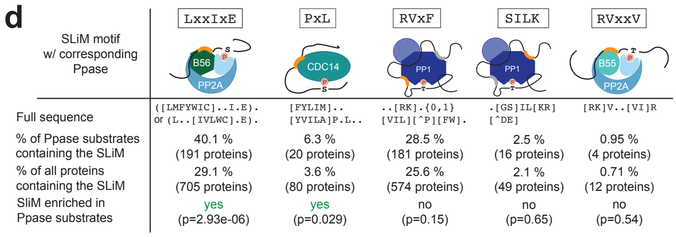

Supplementary Figure 9: CDK-dependent and independent phosphatase substrates

a Motif enrichment analysis of all phosphatase substrates (top panels) and CDK-dependent phosphatase substrates (bottom panels) for kinase motifs using the human kinome atlas[10]. Enrichments were determined using Fisher's exact tests (corrected for multiple testing using the Benjamini-Hochberg method). **b** Motifs used to identify substrates of cell-cycle kinases. **c** Top panel: Bar graph representing the percentage difference of cell cycle kinase substrates present in the phosphatase substrates, compared to all phosphosites. Bottom panel: Bar graph representing the percentage cell cycle kinase substrates present in the phosphatase substrates. **d** Occurrence of SliMs in phosphatase substrates (CDK-dependent and independent)[11–15]. Enrichments were determined using Fisher's exact tests.

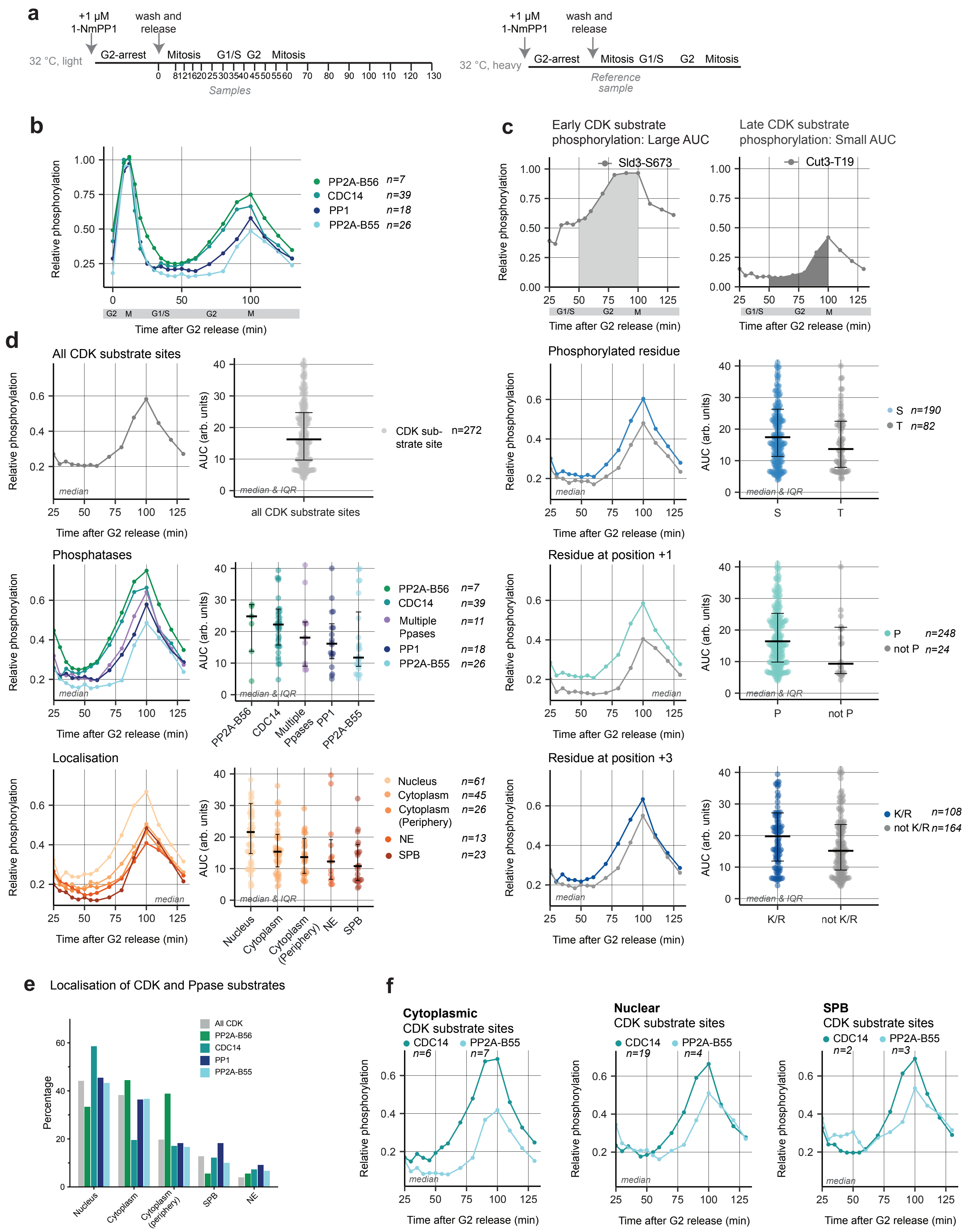

Supplementary Figure 10: Estimating the effect of different determinants on the phosphorylation timing of CDK substrate sites

a Experimental set-up of phosphoproteomic timecourse experiment. A light labelled culture was released from G2 arrest, and protein samples were taken at 20 time points over the first and second cell division cycle. Protein samples were mixed with a common heavy labelled reference, synchronised in mitosis. **b** Median relative phosphorylation of CDK-dependent phosphatase substrates during a synchronised cell cycle, identified by mass spectrometry. Full timecourse

of Figure 4a. **c** Example traces of relative phosphorylation of an example Early (Sld3-S673) and Late (Cut3-T19) CDK substrates throughout a cell cycle. Data are from a synchronised phosphoproteomics timecourse experiment[16]. AUC was calculated as a measure of phosphorylation timing within the cell cycle. **d** Median relative phosphorylation (left) and AUC data (right) of CDK substrate sites during a synchronised cell cycle, split according to opposing phosphatase, localisation of the substrate, phosphorylated residue (S or T), Amino acid at position +3 and amino acid at position +1. Error bars denote the median and IQR. **e** Grouped bar graph of sub-cellular localisations of phosphatase substrates. As a site can have multiple localisations, the percentages may not add up to 100. Fisher's exact tests (corrected for multiple testing using the Benjamini-Hochberg method) were used to test whether phosphatase substrates were significantly enriched in any of the subcellular localisations and showed no significant difference for any of the groups ($p>0.05$). **f** Median relative phosphorylation of CDC14 and PP2A-B55 CDK substrate sites during a synchronised cell cycle, filtered for sites which are localised in the cytoplasm (left panel), nucleus (middle panel) or Spindle pole body (SPB) (right panel).

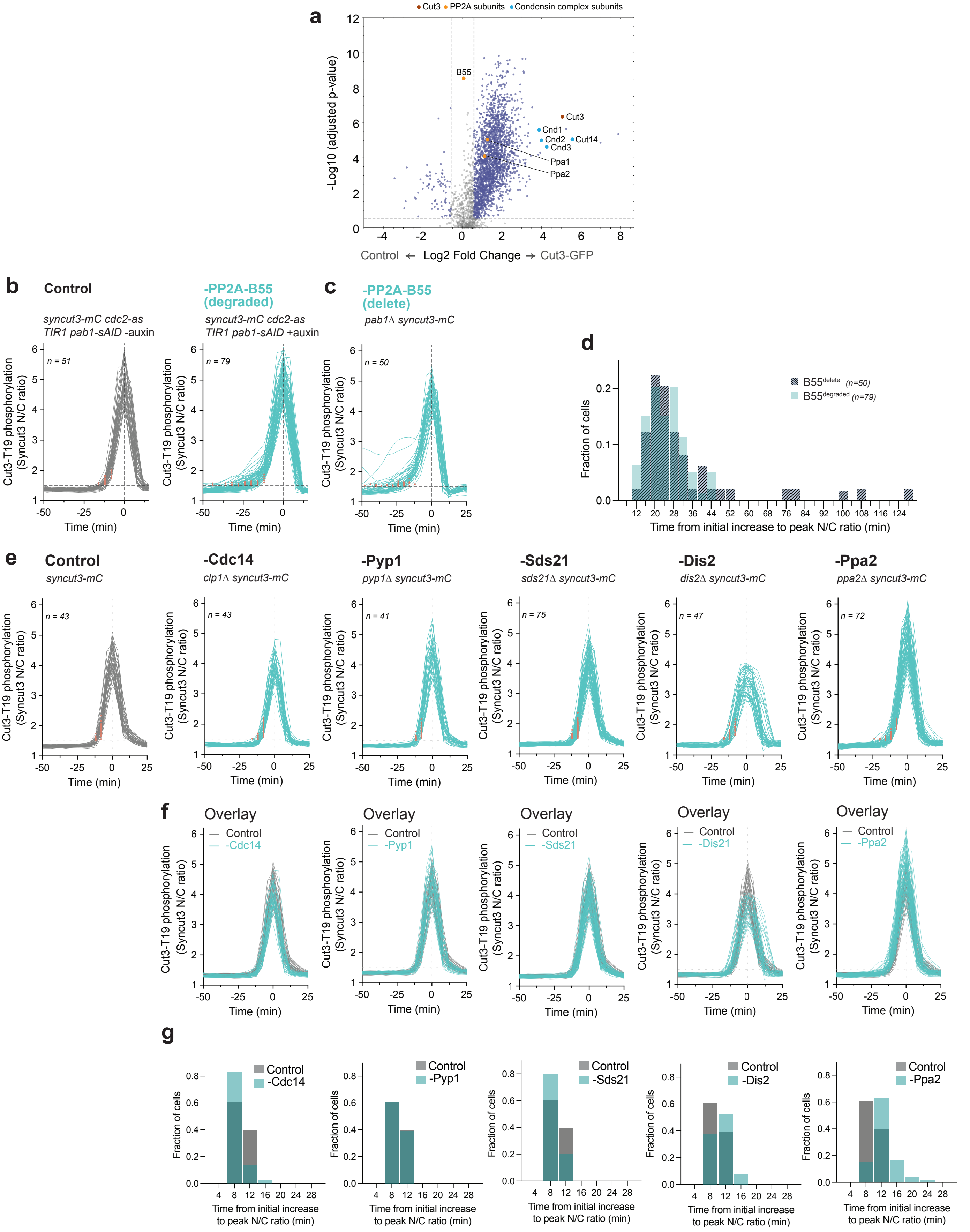

Supplementary Figure 11: Effect of different cell cycle phosphatases on the phosphorylation pattern of SynCyt3

a Volcano plot of comparison between Cut3-GFP and WT control samples (two-tailed Student's t test, FDR 0.01). Proteins labelled in blue showed significant enrichment or depletion in Cut3-GFP co-immunoprecipitation. **b-c** Single-cell traces of SynCut3-mC N/C ratio in the b) presence (control, grey) and absence of PP2A-B55 (B55-degraded, turquoise) and c) B55-deletion (turquoise). Traces are aligned at peak N/C ratio (t=0) and the initial increase in N/C ratio above 1.5 is indicated by orange dots. *n= 51* cells (Control), *n=79* cells (B55-degraded), *n=50* cells (B55-deleted),

representative of *n=3* biological repeats apart from *n=1* for B55-delete. **d** Histograms of time between the initial increase of phosphorylation and peak N/C ratio in the B55-degraded (turquoise) and B55-deleted (dark blue, striped). **e** Single-cell traces of SynCut3-mC N/C ratio in the presence (control, grey) and absence (Ppase-deleted, turquoise) of the indicated phosphatases. Traces are aligned at peak N/C ratio (t=0) and the initial increase in N/C ratio above 1.5 is indicated by orange dots. Cells lacking the major catalytic subunit of PP1 (Dis2) show an aberrant mitotic progression, likely due to a delayed metaphase-anaphase transition (see also Supplementary Fig. 2c-e). *n= 43* cells (Control), *n=43* cells (-CDC14), *n= 41 cells* (-Pyp1), *n= 75 cells* (-Sds21), *n= 47 cells* (-Dis2), *n= 72 cells* (-Ppa2), representative of *n=3* biological repeats. **f** Overlay of the SynCut3-mC N/C traces of control and indicated -Ppase. **g** Histograms of time between the initial increase of phosphorylation and peak N/C ratio in the presence (control, grey) and absence (Ppase-deleted, turquoise) of the indicated phosphatase. This shows that in the absence of the catalytic subunit of PP2A the time between initial and peak Cut3 phosphorylation is longer than in the control. This is not the case in the absence of CDC14 and Sds21, here the time between initial and peak Cut3 phosphorylation is marginally shorter than in the control, likely because global CDK activity is increased in the absence of these phosphatases.

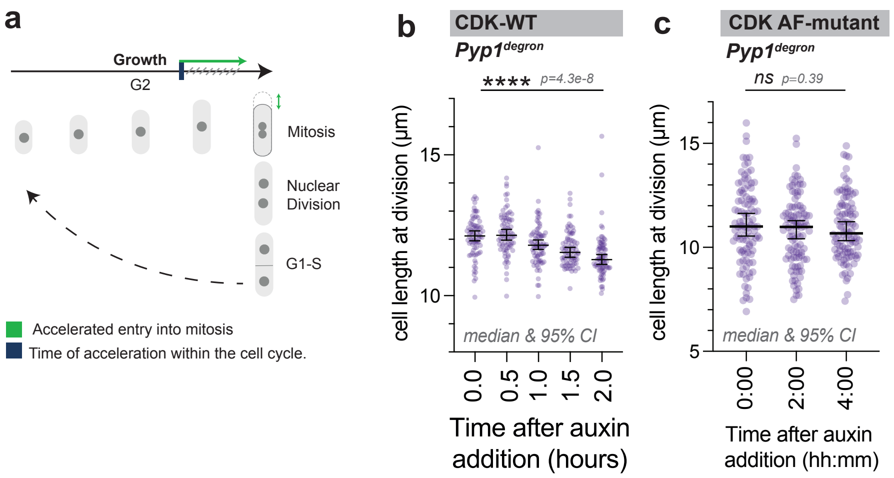

Supplementary Figure 12: Pyp1 delays mitotic onset by affecting CDK activity

a Schematic of cell length extension during *S. pombe* cell cycle and effect of acceleration into mitosis on length at septation. **b-c** Cell length at division at the indicated time after auxin addition of a **b** *Pyp1^{degron}*, *n=80* cells per timepoint, representative of *n=2* biological repeats and **c** *CDK^{AF} Pyp1^{degron}* strain, *n=100* cells per timepoint, representative of *n=3* biological repeats. Error bars represent median and 95% CI. Student's t-test (two-tailed, unpaired) was used to determine statistical difference.

Supplementary references

1. Colaert, N., Helsens, K., Martens, L., Vandekerckhove, J. & Gevaert, K. Improved visualization of protein consensus sequences by iceLogo. *Nat Methods* **6**, 786–787 (2009).
2. Ge, S. X., Jung, D., Jung, D. & Yao, R. ShinyGO: A graphical gene-set enrichment tool for animals and plants. *Bioinformatics* **36**, 2628–2629 (2020).
3. Chica, N., Portantier, M., Nyquist-Andersen, M., Espada-Burriel, S. & Sandra Lopez-Aviles. Uncoupling of Mitosis and Cytokinesis Upon a Prolonged Arrest in Metaphase Is Influenced by Protein Phosphatases and Mitotic Transcription in Fission Yeast. **10**, (2022).
4. Jiang, W. & Hallberg, R. L. Isolation and Characterization of par1+ and par2+: Two Schizosaccharomyces pombe Genes Encoding B′ Subunits of Protein Phosphatase 2A. *Genetics* **154**, 1025–1038 (2000).
5. Clifford, D. M. *et al.* The Clp1/Cdc14 phosphatase contributes to the robustness of cytokinesis by association with anillin-related Mid1. *Journal of Cell Biology* **181**, 79–88 (2008).
6. Mishra, M. *et al.* The Clp1p/Flp1p phosphatase ensures completion of cytokinesis in response to minor perturbation of the cell division machinery in Schizosaccharomyces pombe. *J Cell Sci* **117**, 3897–3910 (2004).
7. Grallert, A. *et al.* A PP1-PP2A phosphatase relay controls mitotic progression. *Nature* **517**, 94–98 (2015).
8. Ohkura, H., Kinoshita, N., Miyatani, S., Toda, T. & Yanagida, M. The fission yeast dis2+ gene required for chromosome disjoining encodes one of two putative type 1 protein phosphatases. *Cell* **57**, 997–1007 (1989).
9. Crooks, G. E., Hon, G., Chandonia, J. M. & Brenner, S. E. WebLogo: A sequence logo generator. *Genome Res* **14**, 1188–1190 (2004).
10. Johnson, J. L. *et al.* An atlas of substrate specificities for the human serine/threonine kinome. *Nature* **613**, 759–766 (2023).
11. Fowle, H. *et al.* Pp2a/b55a substrate recruitment as defined by the retinoblastoma-related protein p107. *Elife* **10**, 1–26 (2021).
12. Hertz, E. P. T. *et al.* A Conserved Motif Provides Binding Specificity to the PP2A-B56 Phosphatase. *Mol Cell* **63**, 686–695 (2016).
13. Kataria, M. *et al.* A PxL motif promotes timely cell cycle substrate dephosphorylation by the Cdc14 phosphatase. *Nat Struct Mol Biol* **25**, 1093–1102 (2018).
14. Hendrickx, A. *et al.* Docking Motif-Guided Mapping of the Interactome of Protein Phosphatase-1. *Chem Biol* **16**, 365–371 (2009).
15. Wakula, P., Beullens, M., Ceulemans, H., Stalmans, W. & Bollen, M. Degeneracy and function of the ubiquitous RVXF motif that mediates binding to protein phosphatase-1. *Journal of Biological Chemistry* **278**, 18817–18823 (2003).
16. Swaffer, M. P., Jones, A. W., Flynn, H. R., Snijders, A. P. & Nurse, P. CDK Substrate Phosphorylation and Ordering the Cell Cycle. *Cell* **167**, 1750-1761.e16 (2016).
