# Supplementary material for: Phosphatase specificity influences phosphorylation timing of CDK substrates during the cell cycle
Source: Nat Commun. 2025 Nov 24;16:11604. doi: 10.1038/s41467-025-66547-5 (PMC12749991; doi:10.1038/s41467-025-66547-5)
Supplement: Supplementary file 2 — Descriptions of Addtional Supplementary Files [file 41467_2025_66547_MOESM2_ESM.pdf]

Description of Additional Supplementary Files

Supplementary Data 1 - List of CDK substrate sites and CDK-dependent phosphatase substrates for PP2A-B55, PP2AB56, CDC14 and PP1. Examples of previously identified phosphatase substrates. List of early phosphorylated CDK substrates and opposing phosphatases.

Supplementary Data 2 - List of CDK-independent sites and CDK-independent phosphatase substrates for PP2A-B55, PP2AB56, CDC14 and PP1.

Supplementary Data 3- List of motifs identified in the phosphatase substrate groups by motif enrichment analysis using the MEME tool suite.

Supplementary Data 4- List of all S. pombe strains used in this study

Supplementary Data 5- List of samples for mass spectrometry experiments

Supplementary Movie 1- Bright field movie, showing arrest of length extension in yeast cells after degradation of PP1 using auxin (PP1-degraded).

Supplementary Movie 2 - Bright field movie, showing growing yeast cells after auxin treatment in the control strain (Control)
