# Supplementary material for: Phosphatase specificity influences phosphorylation timing of CDK substrates during the cell cycle
Source: Nat Commun. 2025 Nov 24;16:11604. doi: 10.1038/s41467-025-66547-5 (PMC12749991; doi:10.1038/s41467-025-66547-5)
Supplement: Supplementary file 10 — Reporting Summary [file 41467_2025_66547_MOESM10_ESM.pdf]

nature portfolio

Corresponding author(s): Theresa Zeisner

Last updated by author(s): Oct 1, 2025

Reporting Summary

Nature Portfolio wishes to improve the reproducibility of the work that we publish. This form provides structure for consistency and transparency in reporting. For further information on Nature Portfolio policies, see our Editorial Policies and the Editorial Policy Checklist.

Statistics

For all statistical analyses, confirm that the following items are present in the figure legend, table legend, main text, or Methods section.

n/a	Confirmed	
☐	☒	The exact sample size (*n*) for each experimental group/condition, given as a discrete number and unit of measurement
☐	☒	A statement on whether measurements were taken from distinct samples or whether the same sample was measured repeatedly
☐	☒	The statistical test(s) used AND whether they are one- or two-sided<br>*Only common tests should be described solely by name; describe more complex techniques in the Methods section.*
☒	☐	A description of all covariates tested
☐	☒	A description of any assumptions or corrections, such as tests of normality and adjustment for multiple comparisons
☐	☒	A full description of the statistical parameters including central tendency (e.g. means) or other basic estimates (e.g. regression coefficient) AND variation (e.g. standard deviation) or associated estimates of uncertainty (e.g. confidence intervals)
☐	☒	For null hypothesis testing, the test statistic (e.g. *F*, *t*, *r*) with confidence intervals, effect sizes, degrees of freedom and *P* value noted<br>*Give P values as exact values whenever suitable.*
☒	☐	For Bayesian analysis, information on the choice of priors and Markov chain Monte Carlo settings
☒	☐	For hierarchical and complex designs, identification of the appropriate level for tests and full reporting of outcomes
☒	☐	Estimates of effect sizes (e.g. Cohen's *d*, Pearson's *r*), indicating how they were calculated

Our web collection on statistics for biologists contains articles on many of the points above.

Software and code

Policy information about availability of computer code

Data collection	Microscopy images were collected using ImageJ 1.52j (NIH).
Data analysis	Mass spectrometry data was searched using MaxQuant (version 1.6.14.0 and 2.4.7.0) and further analysed in Perseus (Version 1.6.14.0). Microscopy images were analysed using ImageJ 1.52j (NIH) and Matlab (2024). All further analysis were done in Matlab, RStudio or GraphPad Prism 10.

For manuscripts utilizing custom algorithms or software that are central to the research but not yet described in published literature, software must be made available to editors and reviewers. We strongly encourage code deposition in a community repository (e.g. GitHub). See the Nature Portfolio guidelines for submitting code & software for further information.

Data

Policy information about availability of data

All manuscripts must include a data availability statement. This statement should provide the following information, where applicable:

- Accession codes, unique identifiers, or web links for publicly available datasets
- A description of any restrictions on data availability
- For clinical datasets or third party data, please ensure that the statement adheres to our policy

All mass spectrometry data generated have been deposited to the ProteomeXchange Consortium via the PRIDE partner repository with dataset identifier PXD060298.

Research involving human participants, their data, or biological material

Policy information about studies with human participants or human data. See also policy information about sex, gender (identity/presentation), and sexual orientation and race, ethnicity and racism.

Reporting on sex and gender	n/a
Reporting on race, ethnicity, or other socially relevant groupings	n/a
Population characteristics	n/a
Recruitment	n/a
Ethics oversight	n/a

Note that full information on the approval of the study protocol must also be provided in the manuscript.

Field-specific reporting

Please select the one below that is the best fit for your research. If you are not sure, read the appropriate sections before making your selection.

☒ Life sciences ☐ Behavioural & social sciences ☐ Ecological, evolutionary & environmental sciences

For a reference copy of the document with all sections, see nature.com/documents/nr-reporting-summary-flat.pdf

Life sciences study design

All studies must disclose on these points even when the disclosure is negative.

Sample size	Sample sizes were not predetermined. We counted 100 cells for determining binucleation indices, at least 80 cells for cell length measurements per strain and timepoint and between 30-80 cells for fluorescent timelapse image analysis for each biological repeat to give an accurate reflection of population behaviour.
Data exclusions	No data was excluded unless otherwise stated.
Replication	All attempts at replication were successful. For phosphoproteomic data, a control strain was used in each experiment giving 4 biological repeats.
Randomization	Randomisation was not relevant to this study, as we only analysed single genotypes for each experiment.
Blinding	For measurements of cell length and analysis of heat-fixed samples comparing different strains, data was analysed blind whenever possible. However, some of the strains displayed obvious phenotypes.

Reporting for specific materials, systems and methods

We require information from authors about some types of materials, experimental systems and methods used in many studies. Here, indicate whether each material, system or method listed is relevant to your study. If you are not sure if a list item applies to your research, read the appropriate section before selecting a response.

Materials & experimental systems

n/a	Involved in the study	
☐	☒	Antibodies
☐	☒	Eukaryotic cell lines
☒	☐	Palaeontology and archaeology
☒	☐	Animals and other organisms
☒	☐	Clinical data
☒	☐	Dual use research of concern
☒	☐	Plants

Methods

n/a	Involved in the study	
☒	☐	ChIP-seq
☐	☒	Flow cytometry
☒	☐	MRI-based neuroimaging

Antibodies

Antibodies used	Primary antibodies: anti-mAID (mouse monoclonal antibody, MBL, M214-3); anti-pTPxK (rabbit monoclonal, anti-phospho-Cdc2-

Antibodies used	Tyr15 (rabbit polyclonal, Cat#9111; RRID: AB_331460) antibody, Cell Signalling Technology, D9V5N); phospho-Cdc2-Tyr15 (rabbit polyclonal, Cat#9111; RRID: AB_331460), phospho-Cut12-pT75 antibody (Grallert et. al., 2013); Cut12 antibody (Grallert et. al., 2013); phospho-Spo15-S121 (Covalab), Spo15-S121 (Covalab). Secondary antibodies: anti-mouse IgG HRP-linked whole antibody from sheep (GE Healthcare Cat# NA931, RRID:AB_772210); anti-rabbit IgG HRP-linked whole antibody from donkey (GE healthcare, Cat# NA934, RRID:AB_772206); sheep IgG HRP-linked whole antibody (Noves, Cat# A16041).
Validation	Antibodies were validated by the manufacturer, Cut12 and Cut12-T75 antibodies were validated in Grallert et. al., 2013

Eukaryotic cell lines

Policy information about cell lines and Sex and Gender in Research

Cell line source(s)	All S. pombe strains used are listed in supplementary table 4.
Authentication	All strains were tested by PCR and positive antibiotic selection, and sequencing where applicable.
Mycoplasma contamination	S. pombe cultures do not get contaminated by mycoplasma.
Commonly misidentified lines (See ICLAC register)	n/a

Plants

Seed stocks	No plants were used in this study.
Novel plant genotypes	n/a
Authentication	n/a

Flow Cytometry

Plots

Confirm that:

☒ The axis labels state the marker and fluorochrome used (e.g. CD4-FITC).

☒ The axis scales are clearly visible. Include numbers along axes only for bottom left plot of group (a 'group' is an analysis of identical markers).

☒ All plots are contour plots with outliers or pseudocolor plots.

☒ A numerical value for number of cells or percentage (with statistics) is provided.

Methodology

Sample preparation	To measure DNA content 1 mL of S. pombe cell culture was pelleted; cells were fixed by addition of 70% v/v ice-cold ethanol and stored at 4 °C. Cells were then pelleted (800 g, 3 min), washed with 50 mM sodium citrate and resuspended in 50 mM sodium citrate containing 0.1 mg/ml RNaseA (Sigma) for 18 hours at 37 °C. 50 mM sodium citrate containing SYTOX Green (Thermo Fisher, final concentration of 1 µM) was added to samples, which were vortexed and sonicated for 10 s per sample (Soniprep 150 plus).
Instrument	BD LSRFortessa cell analyser
Software	FlowJo (v10) was used to analyse flow cytometry data
Cell population abundance	100,000 cells were analysed per strain, per timepoint. Gating for single cells reduced samples down to 70,000-30,000 cells.
Gating strategy	Data was gated for single-cells in Flow-Jo10 based on Side-Scatter-Area vs Forward-Scatter-Area.

☐ Tick this box to confirm that a figure exemplifying the gating strategy is provided in the Supplementary Information.
